# Development of High-Frequency (>60 MHz) Intravascular Ultrasound (IVUS) Transducer by Using Asymmetric Electrodes for Improved Beam Profile

**DOI:** 10.3390/s18124414

**Published:** 2018-12-13

**Authors:** Jin Ho Sung, Jong Seob Jeong

**Affiliations:** Department of Medical Biotechnology, Dongguk University, Seoul 04620, Korea; madeinjinho@dongguk.edu

**Keywords:** high-frequency ultrasound transducer, intravascular ultrasound (IVUS), asymmetric electrode, beam profile, finite element analysis (FEA) simulation

## Abstract

In most commercial single-element intravascular ultrasound (IVUS) transducers, with 20 MHz to 40 MHz center frequencies, a conductive adhesive is used to bond a micro-sized cable for the signal line to the surface of the transducer aperture (<1 mm × 1 mm size) where ultrasound beam is generated. Therefore, the vibration of the piezoelectric layer is significantly disturbed by the adhesive with the signal line, thereby causing problems, such as reduced sensitivity, shortened penetration depth, and distorted beam profile. This phenomenon becomes more serious as the center frequency of the IVUS transducer is increased, and the aperture size becomes small. Therefore, we propose a novel IVUS acoustic stack employing asymmetric electrodes with conductive and non-conductive backing blocks. The purpose of this study is to verify the extent of performance degradation caused by the adhesive with the signal line, and to demonstrate how much performance degradation can be minimized by the proposed scheme. Finite element analysis (FEA) simulation was conducted, and the results show that −3 dB, −6 dB, and −10 dB penetration depths of the conventional transducer were shortened by 20%, 25%, and 19% respectively, while those of the proposed transducer were reduced only 3%, 4%, and 0% compared with their ideal transducers which have the same effective aperture size. Besides, the proposed transducer improved the −3 dB, −6 dB, and −10 dB penetration depths by 15%, 12%, and 10% respectively, compared with the conventional transducer. We also fabricated a 60 MHz IVUS transducer by using the proposed technique, and high-resolution IVUS B-mode (brightness mode) images were obtained. Thus, the proposed scheme can be one of the potential ways to provide more uniform beam profile resulting in improving the signal to noise ratio (SNR) in IVUS image.

## 1. Introduction

It has been well known that the intravascular ultrasound (IVUS) can provide useful diagnostic information, such as lesion location, lumen size, plaque composition, and thickness of the vessel wall by acquiring inside image of the blood vessel [1,2,3,4,5]. In the interventional cardiology using IVUS image, the size of the IVUS transducer is a very critical issue because the catheter combined with the ultrasound transducer must be introduced into the patient’s blood vessel. Another issue is the center frequency of the IVUS transducer. Current commercial IVUS transducers with single-element aperture have center frequencies of 20 to 40 MHz [2,4,6,7,8,9]. In this frequency range, the axial and lateral resolutions are 60~200 μm and 110~400 μm respectively [3,4,7,8,10,11], but those are not sufficient for precise diagnosis. Therefore, there have been lots of efforts to increase the center frequency of the IVUS transducer above 40 MHz, including dual-frequency technique, in order to achieve better spatial resolution [3,4,5,9,12]. As the center frequency is increased, the penetration depth becomes short due to increased attenuation, so the fabrication of high-frequency (>40 MHz) IVUS transducer should be done very carefully to maximize the transmitted and received ultrasound energy [2,3,4,13,14,15,16,17]. Additional issue for the fabrication of the single-element IVUS transducer is how to connect signal and ground electrodes.

In the manufacturing process of the single-element IVUS transducer, the electrode formation can be performed using gold sputtering or wiring process. In the case of the gold sputtering process, a signal line (micro-coaxial cable) is connected to a backing layer by the conductive adhesive, and front side of the IVUS acoustic stack is electrically connected to a metal housing by the gold sputtering process to form a ground electrode [4,18,19]. Alternatively, by using the wiring process, the signal line is attached to the front side of the IVUS acoustic stack, and a ground line is linked to a backing layer. In general, most commercial single-element IVUS transducers use the latter method due to low cost and relatively short production time suitable for mass production compared with the former method. However, in this case, the vibration of the piezoelectric layer is disturbed by the adhesive with the signal line, since a cable for the signal line is bonded to the surface of the piezoelectric layer or conductive matching layer and fixed by the conductive epoxy [7,13,20,21]. That is, considering very small (<1 mm × 1 mm) aperture size of the single-element IVUS transducer, the sensitivity, penetration depth, and beam profile are adversely affected by the adhesive with the signal line. These side effects become more serious as the center frequency of the transducer increases, and the aperture size decreases.

To overcome the aforementioned issues, such as an increase of center frequency more than 40 MHz and suppression of the adhesive region vibration, in this study, a novel IVUS acoustic stack is presented. In the proposed method, asymmetric electrodes are formed below the adhesive region using the conductive and non-conductive backing blocks capable of minimizing the interference effect of the adhesive with the signal line. Finite element analysis (FEA) simulation was conducted to verify the performance of the proposed scheme for a 60 MHz IVUS transducer. To verify how much performance degradation occurs by the adhesive with the signal line, the performance of the conventional and proposed transducers were firstly compared with their ideal transducers which have the same effective aperture size, respectively. Then, a performance comparison was made between the conventional and proposed transducer with their own beam profile. Subsequently, we developed a fabrication process for the proposed technique, and a prototype 60 MHz IVUS transducer was successfully manufactured. The performance was evaluated through electrical impedance, pulse-echo, and B-mode (brightness mode) imaging tests. In Section 2, the basic principle of the proposed method, IVUS transducer design based on FEA, and the fabrication process of the prototype transducer were described. The simulation and experimental results were explained in Section 3. The discussion and conclusion were drawn in Section 4.

## 2. Materials and Methods 

### 2.1. Proposed IVUS Transducer

**Figure 1:**
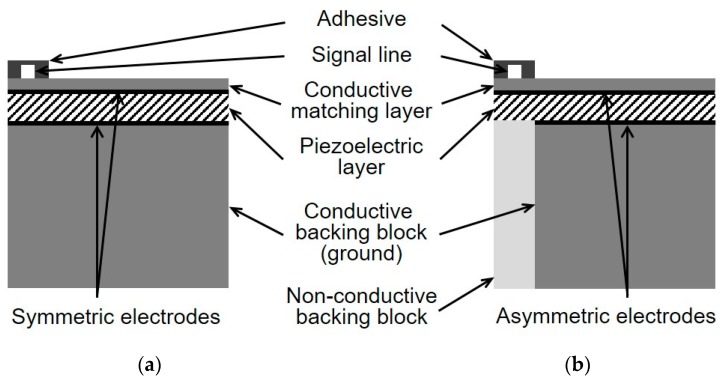
Schematic diagrams of (**a**) conventional transducer; and (**b**) proposed transducer.

Figure 1 shows the schematic diagrams of the conventional and the proposed single-element IVUS transducers. In the case of a conventional transducer (Figure 1a), the adhesive with the signal line is bonded to the surface of the conductive matching layer, and the entire surface, including the adhesive region vibrates when a voltage is applied. On the other hand, in the proposed transducer (Figure 1b), the signal and ground electrodes are not symmetric to each other, since the portion of the ground electrode corresponding to the adhesive region is removed. Besides, unlike the conventional transducer, the backing layer in the proposed scheme is composed of the conductive and non-conductive blocks. Therefore, the adhesive region can be isolated from the applied input voltage by positioning the non-conductive backing block below that region. Consequently, in the proposed technique, the adhesive can connect the signal line with minimized abnormal vibration effect caused by itself.

### 2.2. Finite Element Analysis (FEA) Simulation

**Figure 2:**
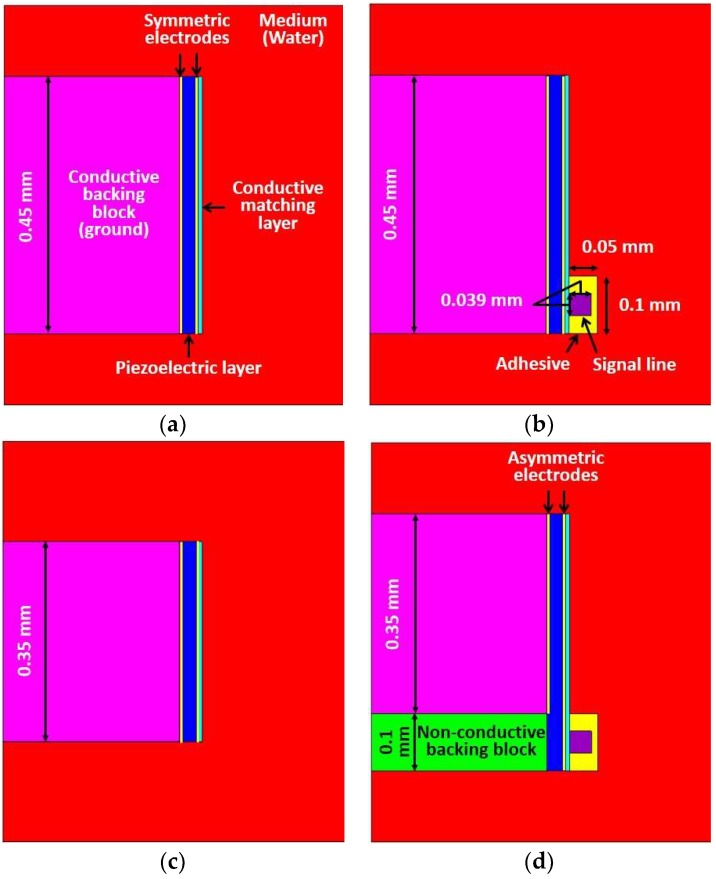
Geometric models of the four designed transducers used for finite element analysis (FEA) simulation: (**a**) Ideal transducer #1; (**b**) conventional transducer; (**c**) ideal transducer #2; and (**d**) proposed transducer.

The performance of the proposed method was demonstrated by FEA based PZFlex program (OnScale, Cupertino, CA, USA). The conventional transducer, the proposed transducer, and two ideal transducers were designed with 60 MHz center frequency. Ideal transducers were used to verify the level of the performance degradation due to the adhesive with the signal line, and -their acoustic stacks were identical to the conventional and proposed transducers respectively, except for the signal line, adhesive, and non-conductive backing block as shown in Figure 2a,c. Although the ideal transducer has no adhesive with the signal line, the electrode to activate the transducer can be virtually formed in the FEA simulation resulting in an ideal situation. The aperture sizes of the ideal transducer #1 and #2 were determined by the activated piezoelectric region, which determined the effective aperture of the conventional and proposed transducers respectively. In other words, the performance of the conventional transducer (Figure 2b) having the conductive adhesive with the signal line was compared with the ideal transducer #1 whose aperture size is identical to that of the conventional transducer, including the adhesive region. In the case of the proposed transducer (Figure 2d), the performance was compared with the ideal transducer #2 whose aperture size is identical to that of the proposed transducer excluding the adhesive region. Therefore, the dimension of the ideal transducer #2 was 0.1 mm smaller than ideal transducer #1. Figure 2 and Table 1 show geometric models and the common design specifications of four transducers used in FEA simulation. Table 2 shows the dimension information of four transducers. 

As a common specification, designed transducers had an aperture size of 0.45 mm width because typical size of an IVUS transducer should be less than 0.5~1 mm to rotate within the catheter tip having 0.9~1.3 mm (2.6~4 F) diameter [3,4,12,13,17,22]. A size of the non-conductive backing block in the proposed scheme was chosen as less than 0.1 × 0.45 mm (L × W) considering both diameters of the signal line in micro co-axial cable (0.039 mm) and the fabrication process. A PMN-32%PT (CTS Corp., Bolingbrook, IL, USA) single crystal was used as a piezoelectric layer, and its properties are shown in Table 3. Silver (Adrich Chem. Co., Milwaukee, WI, USA) loaded epoxy was used as a conductive matching layer, and E-Solder 3022 (Von Roll Isola Inc., New Haven, CT, USA) was used for both adhesive material and backing layer. EPO-TEK 301 (Epoxy technologies, Billerica, MA, USA) was used as a non-conductive backing block in the proposed scheme. A copper was used for a co-axial cable; then 100 V_pp_ negative impulse signal with 5 ns pulse width and two cycles of 60 MHz sinusoidal wave with 50 V_pp_ were used as driving function assuming real situation [23]. The absorption was employed as a boundary condition, and the element size was 1 μm. A stainless steel material was used for a reflector for pulse-echo simulation, and its size was identical to the size of the designed medium size (0.7 mm).

### 2.3. Transducer Fabrication

In FEA simulation, the performance of the proposed transducer was demonstrated compared with the ideal transducer and the conventional transducer. However, the experimental comparison between two types of transducers (conventional and proposed transducers) was not conducted, since the adhesive area on the aperture should be identical to each other. Based on our laboratory equipment, it was very difficult to make two transducers with the same adhesive area over a very small aperture. Instead of that, we applied the proposed technique to 60 MHz prototype transducer to verify the proposed technique can work well or not.

A pre-poled PMN-32%PT with 15 mm × 15 mm size was prepared to fabricate prototype IVUS transducer. Its thickness was about 33 μm for center frequency of 60 MHz, and both sides had electrodes with 1000 Å gold over the chrome seed layer. A mixture of 0.5~1 μm silver particle (Adrich Chem. Co., Milwaukee, WI, USA) and Insulcast 502 (American Safety Technologies, Roseland, NJ, USA) was used for matching layer, and casted on the piezoelectric layer. After that, it was put into a centrifuge for 10 min with 3100 rpm to sink down the particle resulting in desired acoustic impedance (7.3 MRayl) and electrical conductivity. After curing, it was lapped down to 7 μm thickness. With the E-Solder 3022 (5.9 MRayl after centrifuge), the same process was conducted to form a backing layer, and it was lapped until the total thickness of acoustic stack has 0.4 mm. To generate the non-conductive backing block, a dicing machine (DAD 322, Disco Corp., Tokyo, Japan) was employed. A blade with 70 μm width was downed deeply, until it could scratch the surface of PMN-32%PT to partially remove an existing gold electrode resulting in asymmetric electrodes. After that, kerf was filled by EPO-TEK 301 and finished by lapping process to make a flat surface. After dicing job, the final dimension of the fabricated IVUS stack was 0.56 × 0.40 mm (L × W) with 0.07 × 0.40 mm (L × W) exclusive region for the signal line connection. The dimension of the IVUS element was determined by considering the manufacturing capabilities of our laboratory, and thus the electrical impedance at the center frequency was reduced from 50 Ω, which is the ideal impedance matching. To further improve the performance of the proposed transducer, we made the size of the non-conductive backing block 30 μm less than the simulation data. Subsequently, DC 30 kV/cm electric field was applied to the transducer for 10 min at room temperature in air. Note that the above condition was applied to all poling processes, including the pre- and re-poling process. The coaxial cable was connected to the backing and matching layers and fixed by E-Solder 3022. Subsequently, it was put into bullet-type side-looking IVUS metal housing, and additional brass tube was attached to this housing for connecting 50 Ω Sub-Miniature version A (SMA) connector on the other side.

### 2.4. Preparation of Wire and Tissue-Mimic Gelatin Phantoms

The wire phantom was prepared using a tungsten wire with 25 μm diameter. In the case of a tissue-mimic phantom, a brass tube with 5 mm diameter was fixed at the center of a beaker which will be used as a final phantom container, and those were put into a freezer so that phantom hardens quickly. Two different hot plates were prepared, and set to get surface temperature at 130 °C and 110 °C each. Two different beakers with 100 mL de-ionized (DI) water were put on each hot plate and heated for 5~10 min. After that 20 g of gelatin powder (Kraft Foods Inc., Northfield, IL, USA) was dissolved in the beaker on the hot plate having 110 °C surface temperature, and subsequently, the water in the other beaker was also poured into it. 5 min later, 1.3 g, 1.4 g, 1.3 g of 3 μm, 12 μm, and 13 μm aluminum oxide powders (Nanko Abrasive Industry Co., Ltd., Tokyo, Japan), respectively, were added into the solution as a scatterer, and the mixture was held for 40 min while stirring it with a magnetic bar. Then, the surface temperature of the hot plate was gradually cooled down until it was 30 °C, at a rate of approximately 4 °C per minute. Finally, the mixture was poured into the prepared final beaker which was put into the freezer before, and stuffed it in the freezer again for 30 min. After that it was stored in the refrigerator and brass tube was removed before use.

## 3. Results

### 3.1. FEA Simulation Results

#### 3.1.1. Comparison of Electrical Impedance to Ideal Transducers

**Figure 3:**
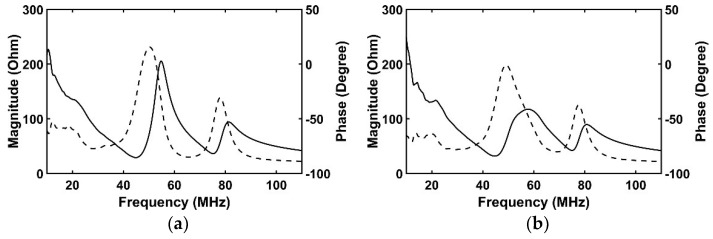

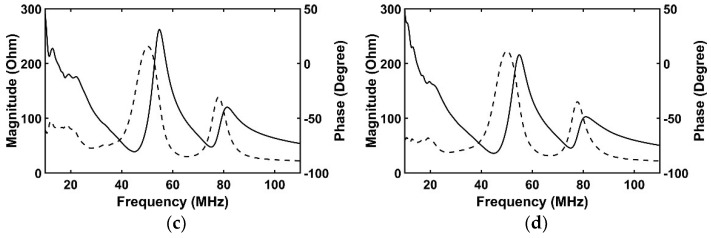
Simulated electrical impedance results of four transducers: (**a**) Ideal transducer #1; (**b**) conventional transducer; (**c**) ideal transducer #2; and (**d**) proposed transducer. The solid line and the dashed line indicate magnitude and phase respectively.

Figure 3 shows the simulated electrical impedance results of four transducers. In the case of the ideal transducer #1 (Figure 3a), the electrical impedances were 28.6 Ω and 200.3 Ω at a resonance frequency of 44.9 MHz and an anti-resonance frequency of 54.7 MHz respectively. The conventional transducer (Figure 3b) has 31.5 Ω at 44.7 MHz and 115.7 Ω at 57.8 MHz. The ideal transducer #2 (Figure 3c) has 38.3 Ω at 44.9 MHz and 254.4 Ω at 54.7 MHz. The proposed transducer has 35.4 Ω at 44.9 MHz and 210.5 Ω at 54.9 MHz as shown in Figure 3d. In comparison with the conventional transducer and ideal transducer #1, the electrical impedance at the resonance frequency was almost similar to each other, but the conventional transducer had very low electrical impedance at anti-resonance frequency compared with the ideal transducer #1. That may be due to the adhesive with the signal line. On the other hand, in the proposed transducer, its electrical impedances at resonance and anti-resonance frequencies were very similar to those of the ideal transducer #2 despite attachment of the adhesive with the signal line. From these results, it was demonstrated that the proposed scheme can effectively suppress the abnormal vibration of the piezoelectric layer generated in the place where the adhesive with the signal line is coupled. Numerically calculated electrical impedance values are described in Table 4.

#### 3.1.2. Comparison of Pulse-Echo to Ideal Transducers

**Figure 4:**
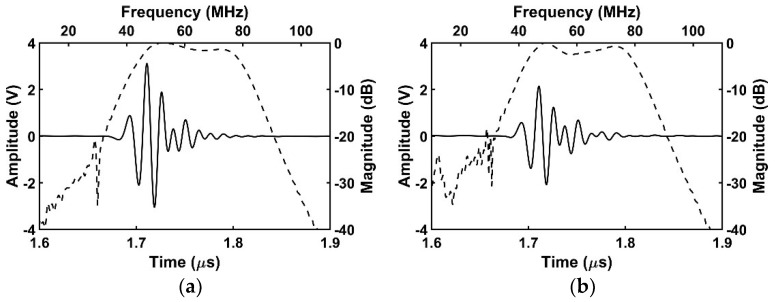

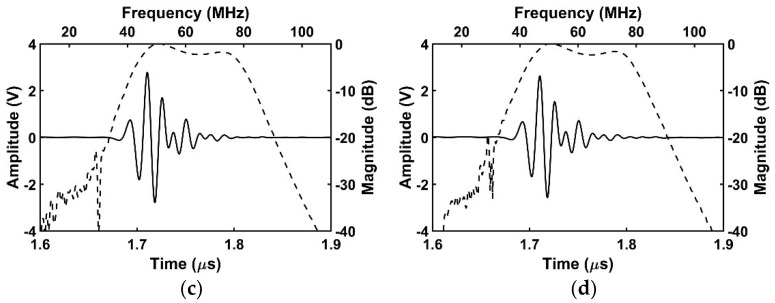
Simulated pulse-echo results of four transducers: (**a**) Ideal transducer #1; (**b**) conventional transducer; (**c**) ideal transducer #2; and (**d**) proposed transducer. The solid line indicates the time-domain response and the dashed line is the frequency-domain response.

In the pulse-echo simulation, the center frequency, and −6 dB fractional bandwidth, were almost maintained as 60.8~61.4 MHz and 63.4~65.7% for all cases as shown in Figure 4. However, regarding an aspect of sensitivity, it was shown that the proposed transducer can minimize the negative effect of the adhesive with the signal line. Calculated peak-to-peak voltages were 6.2 V_pp_, 4.2 V_pp_, 5.6 V_pp_, and 5.2 V_pp_ in order of ideal transducer #1 (Figure 4a), conventional transducer (Figure 4b), ideal transducer #2 (Figure 4c), and proposed transducer (Figure 4d). The ideal transducer #1 and #2 had higher sensitivity than the conventional and proposed transducers respectively, because they had no disturbance by the adhesive with the signal line. In the case of the conventional transducer, the effective aperture size was identical to that of the ideal transducer #1. However, the amplitude of the conventional transducer decreased by 32% because the abnormal vibration of the piezoelectric area below the adhesive region adversely affected the vibration of rest area resulting in degraded echo sensitivity. On the other hand, the proposed transducer had only 7% lower amplitude compared with the ideal transducer #2 whose effective aperture size was identical to the proposed transducer. Additionally, the proposed transducer had a 23% higher amplitude than the conventional one. From these simulation results, it was demonstrated that the proposed transducer can minimize sensitivity degradation caused by the adhesive with the signal line. Table 5 shows the results of a simulated pulse-echo test.

#### 3.1.3. Comparison of Beam Profile to Ideal Transducers

**Figure 5:**
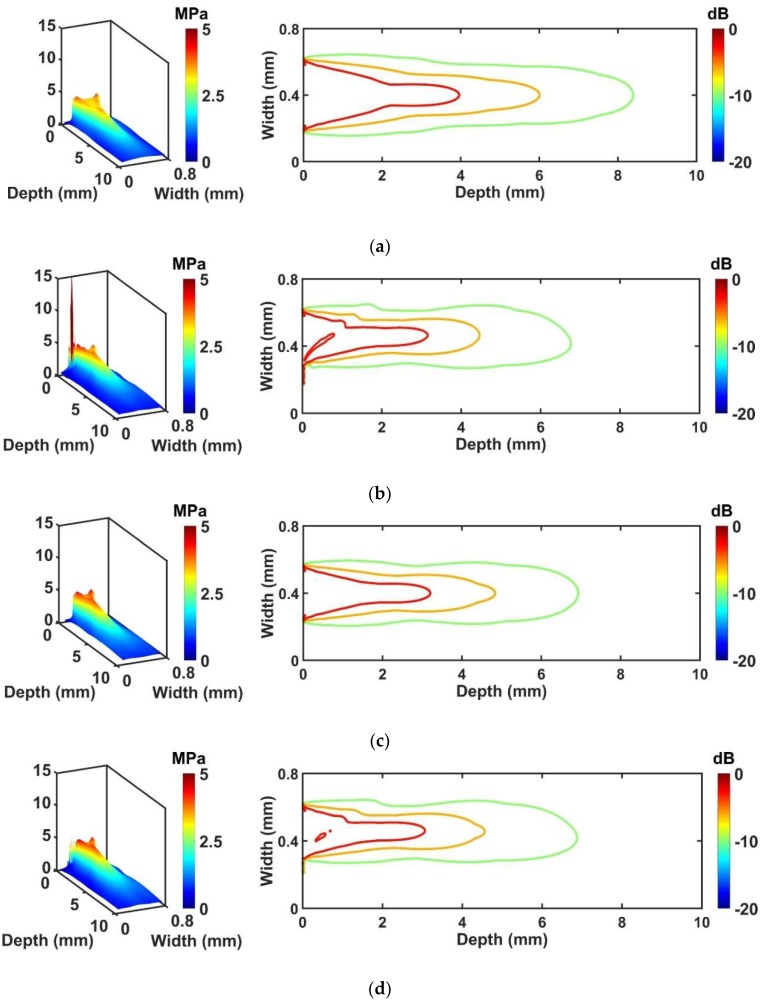
Simulated pressure fields of four transducers: (**a**) Ideal transducer #1; (**b**) conventional transducer; (**c**) ideal transducer #2; and (**d**) proposed transducer. Each contour plot indicates −3 dB, −6 dB, and −10 dB pressure distribution.

The results of pressure field simulation are given in Figure 5. The beam profile was represented by a three-dimensional (3D) graph that clearly shows the pressure distribution inside and outside the wire-connected part. Moreover, a 2D graph shows overall beam distribution within the medium. The purpose of this simulation is to monitor how much performance degradation occurs for the conventional and proposed transducers compared with their ideal transducers. In the case of the conventional transducer (Figure 5b), −3 dB, −6 dB, and −10 dB pressure related to the penetration depth were reduced by 20%, 25%, 19%, respectively, compared with the ideal transducer #1 (Figure 5a). On the other hand, the proposed transducer (Figure 5d) shows only 3%, 4%, and 0% reduced pressure fields for −3 dB, −6 dB, and −10 dB pressure plots respectively, compared with the ideal transducer #2 (Figure 5c). Note that −3 dB, −6 dB, and −10 dB pressure levels of the conventional and proposed transducers were calculated on the basis of results from their ideal transducers. In other words, the real pressure values which are used for determining −3 dB, −6 dB, and −10 dB pressure levels for the ideal transducer #1 and the conventional transducer are identical to each other in order to compare penetration depth fairly. Also, the real pressure values used for determining −3 dB, −6 dB, and −10 dB pressure levels for the ideal transducer #2 and the proposed transducer are identical to each other. Thus, the real pressure values between the conventional and proposed transducers are different in this simulation.

A location where the high-peak pressure occurred as shown in Figure 5b was matched to the adhesive region, including the signal line. This phenomenon may affect the beam distortion of the conventional transducer. In the case of an ideal transducer #1 and #2, general pressure distribution having symmetric beam profile was observed as shown in Figure 5a,c. However, when the adhesive with the signal line is attached, asymmetric beam distribution appeared resulting in reduced penetration depth. This occurs because the wire is bonded to the surface of the piezoelectric region, and this phenomenon becomes worse for the conventional transducer than for the proposed transducer. Table 6 shows the summarized results of pressure field simulation compared with ideal transducers. 

#### 3.1.4. Comparison of Beam Profile between Conventional and Proposed Transducers

**Figure 6:**
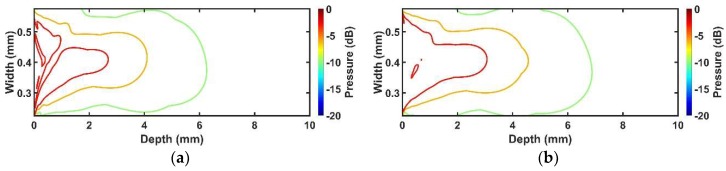
Simulated pressure fields of two transducers: (**a**) Conventional transducer; and (**b**) proposed transducer. Each contour plot indicates −3 dB, −6 dB, and −10 dB pressure distribution.

**Figure 7:**
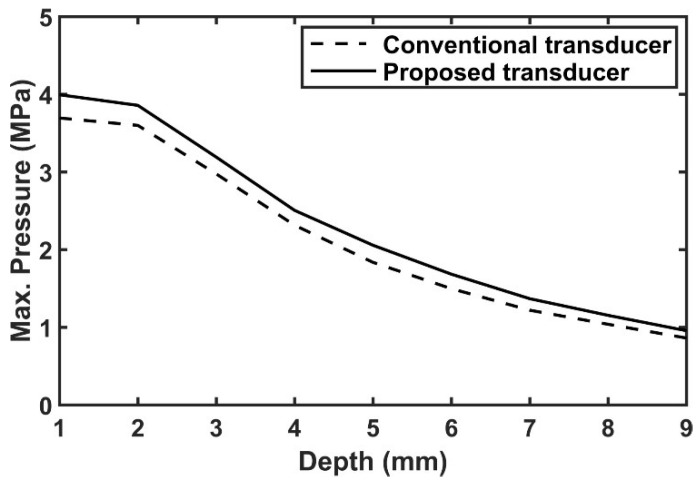
Comparison of simulated pressure pattern for the conventional and proposed transducers.

Figure 5 shows how much the performance of the conventional and proposed transducers is reduced, compared with their ideal transducers. In order to compare the performance between conventional and proposed transducers, their pressure fields were normalized by themselves as shown in Figure 6. Compared with the conventional transducer, the −3 dB, −6 dB, and −10 dB pressure regions of the proposed transducer were extended by 15%, 12%, and 10%, respectively. Additionally, the −3 dB beam profile of the proposed transducer was more evenly distributed, and thus more uniform signal to noise ratio (SNR) can be obtained. Also, the pressure values according to the depth were 7~20% higher than the conventional case as shown in Figure 7. Thus, it can have more extended penetration depth and uniform SNR, as well as higher sensitivity. Table 7 shows the summarized pressure field results of the conventional and proposed transducers.

### 3.2. Experimental Results

**Figure 8:**
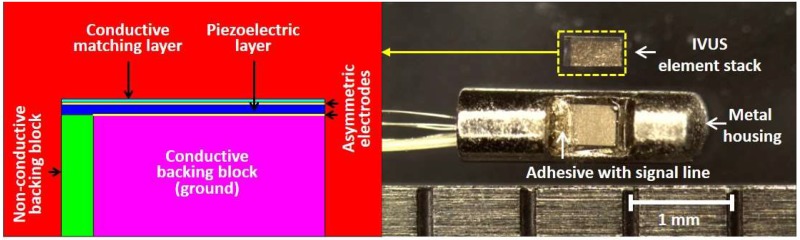
Schematic diagram and photograph of the prototype intravascular ultrasound (IVUS) transducer with the proposed technique: (**left**) Schematic diagram of the IVUS element stack; and (**right**) photograph of the (top) fabricated IVUS element stack with side view; and (bottom) fully fabricated IVUS transducer at proximal portion before combining a needle type brass tube.

**Figure 9:**
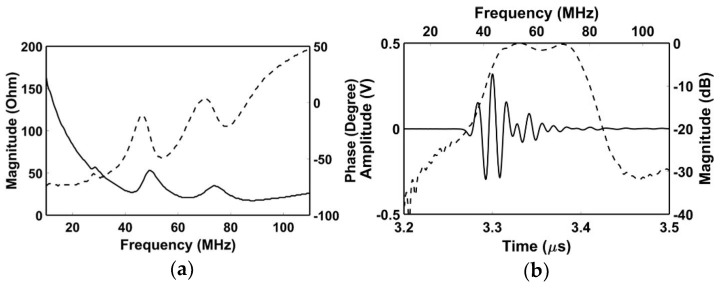
Measured results of the proposed IVUS transducer: (**a**) Electrical impedance (solid line—magnitude; dashed line—phase); and (**b**) pulse-echo response (solid line—time-domain response, dashed line—frequency-domain response).

Figure 8 shows a photograph of the fabricated IVUS acoustic stack and transducer by using the proposed scheme. To evaluate the performance of the transducer, electrical impedance measurement was firstly conducted in the air by using an impedance analyzer (4294A, Agilent Technologies, Santa Clara, CA, USA), and subsequently pulse-echo test was performed. A quartz was used as a target which is located at 2.5 mm away from the surface of the transducer considering effective aperture size of 0.49 × 0.40 mm (L × W) after the signal line connection. A pulser/receiver system (UT340, UTEX Scientific Instruments Inc., Mississauga, ON, Canada) applied 100 V_pp_ negative impulse signal with 5 ns pulse width to the transducer, and received the echo signal. Note that the received gain was set to 0 dB to minimize amplifier noise. Then, the pule-echo signal was displayed by a digital oscilloscope (DSO-X 3034A, Agilent Technologies, Santa Clara, CA, USA). Figure 9 shows the measured electrical impedance and pulse-echo results for the fabricated IVUS transducer. There were two resonance peaks due to one matching layer, and electrical impedance was measured at 23 Ω at 60 MHz. The center frequency, and −6 dB fractional bandwidth, were 60.2 MHz and 57.2%, respectively. The amplitude of the received signal was measured at 0.62 V_pp_ with 0 dB received gain which is sufficient to detect lesion within the vessel.

**Figure 10:**
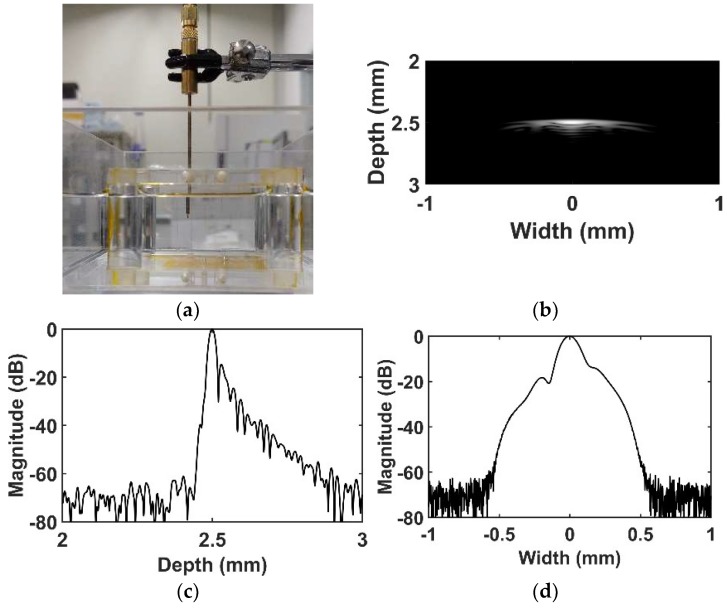
Experimental setup and B-mode image about the point spread function of a wire phantom by using the proposed IVUS transducer: (**a**) Experimental setup; (**b**) B-mode image; (**c**) axial beam pattern; and (**d**) lateral beam pattern.

Figure 10 shows an experimental setup and measured IVUS B-mode image of a wire phantom to measure spatial resolution based on experimental point spread function. A tungsten wire with 25 μm diameter was located at a natural focal depth of 2.5 mm. To acquire image, a motor and motor-controller (SHOT-304GS, SIGMA KOKI, Tokyo, Japan), data acquisition (DAQ) board (CS121G2, GaGe Applied Technologies Inc., Lachine, QC, Canada), and pulser/receiver system were used under the control of LABView program (National Instruments, Austin, TX, USA). A 100 V_pp_ negative impulse signal with 5 ns pulse width was used for the input signal. Herein, linear scanning was employed with 2 μm step size. A received gain was 20 dB, and a logarithmic compression with 40 dB dynamic range was applied. To remove unwanted noise, 128-order finite impulse response (FIR) band pass filter with cutoff frequency 30~90 MHz was used. The −6 dB axial and −6 dB lateral beam widths were measured at 24.8 μm and 156.1 μm respectively, from the point spread function of a wire phantom.

**Figure 11:**
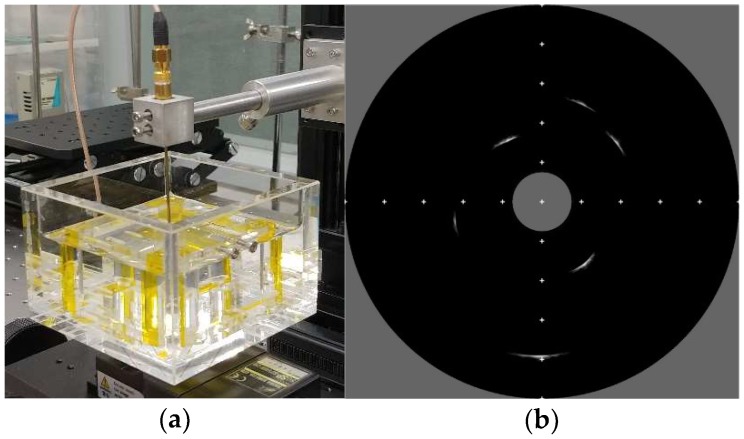

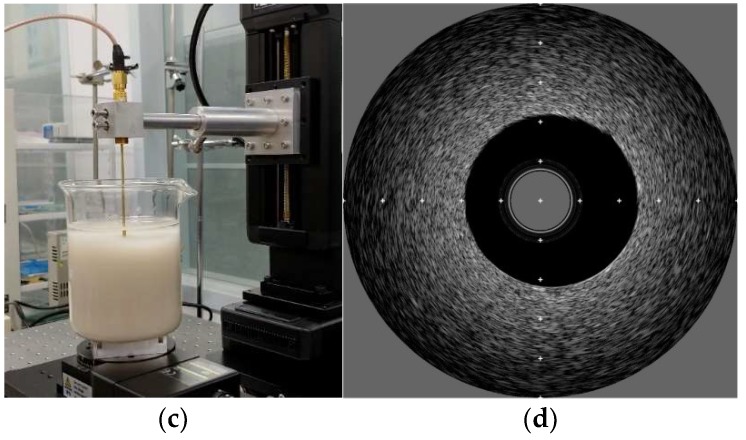
Experimental setups and 360° rotated B-modes images for the proposed IVUS transducer: (**a**) Experimental setup for wire phantom; (**b**) B-mode image of wire phantom; (**c**) experimental setup for tissue-mimic gelatin phantom; and (**d**) B-mode image of tissue-mimic gelatin phantom.

For further performance evaluation, 360° rotated B-mode image was obtained using the tungsten wire phantom with 25 μm diameter and tissue-mimic phantom. The experimental setup, including equipment, received gain, filter condition, and dynamic range of the logarithmic compression was identical to experimental setup for previous wire phantom experiment except that rotary motor was used with 0.5° step size. Figure 11 shows an experimental setup and acquired 360° rotated IVUS B-mode images for wire and tissue-mimic gelatin phantoms, and the marks in B-mode images indicate 1 mm interval.

## 4. Discussion and Conclusions

In this study, a modified structure of an IVUS transducer was proposed capable of improving the performance of the single-element IVUS transducer. Most commercial IVUS transducers with single-element type have suffered from relatively reduced sensitivity, shortened penetration depth, and distorted beam profile compared with the ideal transducer because the conductive adhesive with the signal line is coupled on the surface of the piezoelectric layer or conductive matching layer where ultrasound beam is generated. In the proposed scheme, the adhesive region for wiring can be isolated from the activated piezoelectric region by positioning the non-conductive backing block below the asymmetric electrodes. In other words, the conductive adhesive can connect the signal line to a piezoelectric layer with minimized vibration effect, and thus ultrasound beam in this region is hardly generated. As a result, that region can be utilized as an exclusive region for the signal line connection. To demonstrate the performance of the proposed scheme, FEA simulation was conducted, and its feasibility was verified by fabrication of prototype IVUS transducer.

The FEA simulations for performance comparison were conducted by designing four kinds of transducers. Two ideal transducers with different aperture size were employed to compare the performance of the conventional and proposed transducers. - Considering the activated region size of the piezoelectric layer except for the adhesive region, the piezoelectric area of the ideal transducer #2 was smaller than ideal transducer #1. The electrical impedance and pulse-echo results of the proposed transducer were similar to ideal transducer #2 which means that the proposed structure will operate normally. For the conventional transducer, the amplitude of the pulse-echo signal was 32% lower, and the electrical impedance pattern was different from the ideal transducer #1, and these results influenced the pressure profile simulation. When we compare the propagation performance of the proposed transducer to ideal transducer #2, the reduced penetration depth was less than 4%. However, the conventional transducer shows a reduction of the penetration depth as less than 25%, compared with the ideal transducer #1. 

In the case of the comparison between conventional and proposed cases, the proposed transducer had 10~15% improved penetration depth. The pressure of the proposed transducer was 7~20% higher than the conventional transducer, as well as more uniform pressure distribution. The reason for these results can be explained by acoustic damping effect and energy distribution. When the adhesive with the signal line was applied, it makes somehow vibration of the piezoelectric layer to be suppressed, so conventional transducer is bound to be affected from it more than the proposed transducer because it is attached on the surface where ultrasound beam is generated. In addition, a large amount of energy originated below the adhesive region was concentrated on the adhesive with the signal line, and thus the ultrasound energy traveling into the medium was reduced resulting in distortion of the beam profile. Whereas, in the proposed transducer, since the adhesive with the signal line is applied on the non-activated piezoelectric region, damping effect is not large unlike the conventional transducer, and energy was scarcely concentrated on the adhesive region resulting in minimized performance degradation. Thus, the proposed structure can minimize undesired effect, such as degradation of sensitivity, shortened penetration depth, and distortion of beam profile caused by the adhesive with the signal line on the surface of the transducer. According to the pressure field simulation, it was shown that the attachment of the adhesive with the signal line caused severe beam distortion resulting in asymmetric beam profile with a shortened penetration depth. This can cause image degradation, such as distorted target image and decrease of SNR. However, the simulation results show that the proposed transducer can alleviate the level of the aforementioned side effect although it also shows a slightly asymmetric pattern.

In order to experimentally verify whether the proposed transducer can work normally or not, the prototype transducer using the proposed technique was fabricated. The center frequency was 60 MHz and the size of the dedicated adhesive region was smaller than simulation data resulting in increased fabrication difficulty but expecting improved performance. The −6 dB axial and lateral beam widths were higher than 40 MHz IVUS transducer. Both wire phantom and tissue-mimic phantom B-mode images were successfully obtained. 

About the proposed technique, the size of the non-conductive region does not need to be wide. In our experiment, that size is 12.5% of the total aperture, this value may not affect the vibration mode of the piezoelectric layer. The effective aperture size of the proposed transducer is reduced compared with the conventional transducer. However, the proposed transducer shows more normal electrical impedance pattern, high sensitivity, and broader penetration depth. In other words, although the effective aperture size of the conventional transducer is large, the adhesive with the signal line occupies that increased area, thereby reducing the penetration depth of the ultrasonic wave. The symmetric beam profile was distorted even though the proposed technique was applied, but that was not much compared with the conventional transducer. That is, the aforementioned issues will be generated both the conventional and proposed transducer, but the proposed transducer will show much better performance compared with the conventional transducer.

In this paper, the performance of the proposed transducer was not experimentally compared with the conventional transducer. For fair performance comparison between conventional and proposed transducers, the adhesive area which is a critical factor to determine the performance should be identical to each other. However, based on our laboratory equipment, it was very difficult to make two transducers with the same adhesive area over a very small aperture. That job should be done manually resulting in human error. Thus, we had to compare the performance by using FEA simulation. Instead of performance comparison, we fabricated a prototype transducer to show that the proposed technique works well. In our experimental results, a wire phantom and tissue-mimic phantom images were obtained successfully.

In this study, we proposed an alternate method of fabricating high-frequency (60 MHz) single-element IVUS transducer in order to reduce the pressure distortion caused by the conductive adhesive with the signal line. The proposed technique suggests assigning a dedicated region for the wiring based on asymmetric electrodes, which is not a part of the active region of the piezoelectric layer. The non-conductive backing block was located under that region, and thus abnormal vibration can be minimized. That is to say, distortion of the ultrasound beam profile and the consequent reduction of sensitivity and penetration depth can be minimized. The FEA simulation and experimental results using the prototype IVUS transducer with 60 MHz center frequency show that the proposed technique can be one of the useful ways to improve SNR of high-frequency IVUS image.

## Figures and Tables

**Table 1:** Common design specifications of four transducers used in FEA simulation.

Center frequency [MHz]	60
Thickness of the piezoelectric layer [mm]	0.033
Thickness of the matching layer [mm]	0.007
Thickness of the backing layer [mm]	0.308
Piezoelectric material	PMN-32%PT
Matching layer material	Silver loaded epoxy
Backing layer material	E-Solder 3022
Adhesive material	E-Solder 3022
Signal line material	Copper

**Table 2:** Dimension information of four transducers used in FEA simulation.

Transducers	Total Piezoelectric Region [mm]	Effective Aperture [mm]
Ideal transducer #1 (L × W)	0.45 × 0.45	0.45 × 0.45
Conventional transducer (L × W)	0.45 × 0.45	0.45 × 0.45
Ideal transducer #2 (L × W)	0.35 × 0.45	0.35 × 0.45
Proposed transducer (L × W)	0.45 × 0.45	0.35 × 0.45

**Table 3:** Material properties of PMN-32%PT used in FEA simulation.

Elastic stiffness c^E^ (10^10^ N/m^2^)	c_11_	11.21
	c_12_	10.16
	c_13_	9.04
	c_33_	9.68
	c_44_, c_55_	6.05
	c_66_	5.51
Mechanical Q	50	
Dielectric constant (ε^S^/ε_0_)	ε_11_	1368
	ε_33_	700
Piezoelectric stress e (C/m^2^)	e_13_	−15.99
	e_33_	19.42
	e_15_	8.69
Density (kg/m^3^)	*ρ*	8100

**Table 4:** Simulated electrical impedance results.

	Resonance Frequency	Anti-Resonance Frequency
Ideal transducer #1	28.8 Ω @ 44.9 MHz	205.3 Ω @ 54.7 MHz
Conventional transducer	31.7 Ω @ 44.7 MHz	117.5 Ω @ 57.8 MHz
Ideal transducer #2	38.7 Ω @ 44.9 MHz	262.1 Ω @ 54.7 MHz
Proposed transducer	35.7 Ω @ 44.9 MHz	215.8 Ω @ 54.9 MHz

**Table 5:** Simulated pulse-echo test results.

	Center Frequency (MHz)	−6 dB Bandwidth (%)	Amplitude (V_pp_)
Ideal transducer #1	61.4	63.6	6.2
Conventional transducer	61.2	65.7	4.2
Ideal transducer #2	61.2	63.4	5.6
Proposed transducer	60.8	63.7	5.2

**Table 6:** Penetration depth calculation by using pressure field simulation results compared with ideal transducers.

Transducers	Maximum Penetration Depth (mm)			Δ Penetration Depth (%)		
	−3 dB	−6 dB	−10 dB	−3 dB	−6 dB	−10 dB
Ideal transducer #1	4.0	6.0	8.4	-	-	-
Conventional transducer	3.2	4.5	6.8	−20	−25	−19
Ideal transducer #2	3.2	4.8	6.9	-	-	-
Proposed transducer	3.1	4.6	6.9	−3	−4	0

**Table 7:** Penetration depth calculation based on pressure field simulation results of the conventional and proposed transducers.

Transducers	Maximum Penetration Depth (mm)			Δ Penetration Depth (%)		
	−3 dB	−6 dB	−10 dB	−3 dB	−6 dB	−10 dB
Conventional transducer	2.7	4.1	6.3	-	-	-
Proposed transducer	3.1	4.6	6.9	15	12	10

## References

[1] Katouzian A., Angelini E.D., Carlier S.G., Suri J.S., Navab N., Laine A.F. (2012). A state-of-the-art review on segmentation algorithms in intravascular ultrasound (IVUS) images. IEEE Trans. Inf. Technol. Biomed..

[2] Katouzian A., Angelini E., Lorsakul A., Sturm B., Laine A.F. Lumen border detection of intravascular ultrasound via denoising of directional wavelet representations. Proceedings of the 5th International Conference on Functional Imaging and Modeling of the Heart (FIMH).

[3] Foster F.S., Pavlin C.J., Harasiewicz K.A., Christopher D.A., Turnbull D.H. (2000). Advances in ultrasound biomicroscopy. Ultrasound Med. Biol..

[4] Li X., Wu W., Chung Y., Shih W.Y., Shih W., Zhou Q., Shung K.K. (2011). 80-MHz intravascular ultrasound transducer using PMN-PT free-standing film. IEEE Trans. Ultrason. Ferroelectr. Freq. Control.

[5] Prati F., Arbustini E., Labellarte A., Bello B.D., Sommariva L., Mallus M.T., Pagano A., Boccanelli A. (2001). Correlation between high frequency intravascular ultrasound and histomorphology in human coronary arteries. Heart.

[6] Fujii K., Hao H., Shibuya M., Imanaka T., Fukunaga M., Miki K., Tamaru H., Sawada H., Naito Y., Ohyanagi M. (2015). Accuracy of OCT, grayscale IVUS, and their combination for the diagnosis of coronary TCFA: An ex vivo validation study. JACC Cardiovasc. Imaging.

[7] Ma T., Yu M., Chen Z., Fei C., Shung K.K., Zhou Q. (2015). Multi-frequency intravascular ultrasound (IVUS) imaging. IEEE Trans. Ultrason. Ferroelectr. Freq. Control.

[8] Ma J., Martin K.H., Li Y., Dayton P.A., Shung K.K., Zhou Q., Jiang X. (2015). Design factors of intravascular dual frequency transducers for super-harmonic contrast imaging and acoustic angiography. Phys. Med. Biol..

[9] Qiu W., Chen Y., Wong C., Liu B., Dai J., Zheng H. (2015). A novel dual-frequency imaging method for intravascular ultrasound applications. Ultrasonics.

[10] Elliott M.R., Thrush A.J. (1996). Measurement of resolution in intravascular ultrasound images. Physiol. Meas..

[11] Brezinski M.E., Tearney G.J., Weissman N.J., Boppart S.A., Bouma B.E., Hee M.R., Weyman A.E., Swanson E.A., Southern J.F., Fujimoto J.G. (1997). Assessing atherosclerotic plaque morphology: Comparison of optical coherence tomography and high frequency intravascular ultrasound. Heart.

[12] Yuan J., Rhee S., Jiang X.N. 60 MHz PMN-PT based 1-3 composite transducer for IVUS imaging. Proceedings of the 2008 IEEE Ultrasonics Symposium.

[13] Vos H.J., Frijlink M.E., Droog E., Goertz D.E., Blacquière G., Gisolf A., Jong N.D., Van der Steen A.F.W. (2005). Transducer for harmonic intravascular ultrasound imaging. IEEE Trans. Ultrason. Ferroelectr. Freq. Control.

[14] Cannata J.M., Ritter T.A., Chen W., Silverman R.H., Shung K.K. (2003). Design of efficient, broadband single-element (20–80 MHz) ultrasonic transducers for medical imaging applications. IEEE Trans. Ultrason. Ferroelectr. Freq. Control.

[15] Mamou J., Ketterling J.A., Silverman R.H. (2008). Chirp-coded excitation imaging with a high-frequency ultrasound annular array. IEEE Trans. Ultrason. Ferroelectr. Freq. Control.

[16] Fei C., Chiu C.T., Chen X., Chen Z., Ma J., Zhu B., Shung K.K., Zhou Q. (2016). Ultrahigh frequency (100 MHz–300 MHz) ultrasonic transducers for optical resolution medical imaging. Sci. Rep..

[17] Mamou J., Aristizábal O., Silverman R.H., Ketterling J.A., Turnbull D.H. (2009). High-frequency chirp ultrasound imaging with an annular array for ophthalmologic and small-animal imaging. Ultrasound Med. Biol..

[18] Yan X., Lam K.H., Li X., Chen R., Ren W., Ren X., Zhou Q., Shung K.K. (2013). Lead-free intravascular ultrasound transducer using BZT-50BCT ceramics. IEEE Trans. Ultrason. Ferroelectr. Freq. Control.

[19] Yoon S., Williams J., Kang B.J., Yoon C., Cabrera-Munoz N., Jeong J.S., Lee S.G., Shung K.K., Kim H.H. (2015). Angled-focused 45 MHz PMN-PT single emenet transducer for intravascular ultrasound imaging. Sens. Actuators A.

[20] Thomas L.J., Gilmore R.S., Trzaskos C.R. (1990). High Frequency Focused Ultrasonic Transducer for Invasive Tissue Characterization. U.S. Patent.

[21] Jian X., Han Z., Liu P., Xu J., Li Z., Li P., Shao W., Cui Y. (2017). A high frequency geometric focusing transducer based on 1–3 piezocomposite for intravascular ultrasound imaging. BioMed Res. Int..

[22] Kern M.J., Sorajja P., Lim M.J. (2018). Intravascular Lesion Assessment: Physiology and Imaging. Interventional Cardiac Catheterization Handbook.

[23] Sridharan A., Eisenbrey J.R., Machado P., deMuinck E.D., Forsberg F. (2013). Delineation of atherosclerotic plaque using subharmonic imaging filtering techniques and a commercial intravascular ultrasound system. Ultrason. Imaging.

